# RETROSPECTIVE LIFE HISTORY SURVEY REVEALED AUTOBIOGRAPHICAL MEMORY BUMP

**DOI:** 10.1093/geroni/igz038.2559

**Published:** 2019-11-08

**Authors:** Marina Larkina, Lindsey Meister, Jacqui Smith

**Affiliations:** 1 Institute for Social Research, University of Michigan, Ann Arbor, Michigan, United States; 2 University of Michigan, Ann Arbor, Michigan, United States

## Abstract

The reminiscence bump is a well-documented autobiographical memory phenomenon characterized by middle-aged and older adults reporting a disproportionate number of memories from adolescence and early adulthood (Rubin, Wetzler, & Nebes, 1986). It is typically assessed through either cue word or important memory techniques. The Life History Mail Survey (LHMS) in the Health and Retirement Study affords unique data to investigate this phenomenon among a representative US sample of older adults. At the beginning of the LHMS, participants (N=3088, M age=70, range 50-107) completed a calendar noting the important things that happened to them in seven life decades, starting with ages 0-9 and ending by ages 70-79 (or their actual age). For each life period, we coded the number of events respondents reported. We observed significantly more memories reported for the age decade 20-29, compared with other life periods (80% vs 47-66%). Our results are consistent with previous findings in the autobiographical memory literature. Follow-up analyses evaluated existing theoretical accounts of the bump, such as cultural life script theory which suggests that life events occur in a specific order and are characterized by a prototypical life course. For example, we determined whether respondents’ sociodemographic characteristics, such as age cohort, gender, marital and educational histories (information available in LHMS) influenced the size and temporal location of the reminiscence bump. We also analyzed the content of reported important life events to investigate whether types of events included in each decade of life are consistent with the cultural life script account of the phenomenon.

